# Quality differentiation and salidroside biosynthesis in *Rhodiola crenulata*, *R. fastigiata*, and intergrades

**DOI:** 10.3389/fpls.2026.1873808

**Published:** 2026-06-29

**Authors:** Chuanxin Mo, Qiuling Wang, Binfan Si, Mingyue Yan, Cunhua Zeng, Youjun Lu, Dongsheng Ren, Wenshuai Chen, Zhen Ni, Hua Chen, Binjie Xu, Ma Yu, Jianhe Wei

**Affiliations:** 1 College of Life Sciences and Agri-forestry, Southwest University of Science and Technology, Mianyang, Sichuan, China; 2 Key Laboratory of Bioactive Substances and Resources Utilization of Chinese Herbal Medicine, Ministry of Education and National Engineering Laboratory for Breeding of Endangered Medicinal Materials, Institute of Medicinal Plant Development, Chinese Academy of Medical Sciences and Peking Union Medical College, Beijing, China; 3 Tibet Rhodiola Pharmaceutical Holding Co., Lhasa, Xizang, China; 4 Institute of Plateau Biology of Xizang Autonomous Region, Lhasa, Xizang, China; 5 Innovative institute of Chinese Medicine and Pharmacy, Chengdu University of Traditional Chinese Medicine, Chengdu, Sichuan, China

**Keywords:** anatomical, hybrids, molecular docking, *Rhodiola crenulata*, salidroside, UDP-glycosyltransferases

## Abstract

*Rhodiola crenulata*, the only authentic medicinal species recorded in the Chinese Pharmacopoeia, is increasingly threatened by resource depletion and adulteration, raising concerns regarding its quality and safety. Here, an integrative multi-analysis combining phenotypic traits, anatomical characteristics, metabolite profiling, and transcriptome sequencing was conducted to systematically compare *R. crenulata*, *R. fastigiata*, and naturally occurring intergrades. *R. crenulata* exhibited a lower rhizome yield (34.9 g) than *R. fastigiata* (55.6 g) and intergrades (39.5 g), but displayed a larger stem diameter (3.9 mm) and leaf width (1.3 cm), along with a shorter stem length (14.8 cm). Anatomically, it possessed a double-layered vascular cambium with an irregular arrangement, accompanied by significantly larger vessel elements (324.2 μm²) and storage parenchyma cells (1882.5 μm²). Metabolite analysis showed that the salidroside content in *R. crenulata* (0.81%) was 20-fold higher than in *R. fastigiata* and 4-fold higher than in intergrades, while tyrosol (0.45‰) and total flavonoids (7.2%) remained the highest. Transcriptomic analysis revealed extensive differential gene expression among genotypes and along different axial positions of the rhizome, with the phenylpropanoid biosynthesis and tyrosine metabolism pathways being significantly enriched. Co-expression and molecular docking identified six candidate UDP-glycosyltransferases with strong in silico substrate-binding affinity, among which *RcUGT5914* showed species-specific expression. Collectively, this research provides a multi-dimensional framework for the accurate authentication and quality evaluation of *R. crenulata*, and establishes a molecular basis for its conservation and genetic improvement.

## Highlights

- A multi-dimensional framework for authenticating R. *crenulata* and evaluating its quality
- *R. crenulata* accumulates 20-fold higher salidroside than *R. fastigiata* and possesses distinctive anatomical traits
- Axial-position transcriptomics reveal spatial regulation of key biosynthetic pathways
- RcUGT5914 is a species-specific UDP-glycosyltransferase with strong in silico substrate-binding affinity

## 1 Introduction

The genus *Rhodiola* (Crassulaceae) comprises approximately 136 species worldwide, with over 70 taxa distributed across China, predominantly on the Qinghai–Tibet Plateau and adjacent high-altitude regions (2000–5600 m) (Kelly, 2001; Tao et al., 2019; Liu et al., 2025). These alpine perennial herbs are well adapted to extreme environments and have long been utilized in traditional medicine for their adaptogenic, anti-fatigue, and anti-hypoxia properties (Khanum et al., 2005; Tinsley et al., 2024). Among them, *Rhodiola crenulata* is recognized as the only authentic medicinal source listed in the *Chinese Pharmacopoeia* (2025). However, wild *R. crenulata* exhibits extremely low yield and an exceptionally long growth cycle, often exceeding 30 years (**Supplementary Figure 1A**). Coupled with the long-standing practice of harvesting entire underground parts, these factors have driven a sharp decline in wild populations, and the species has been designated a national Class II protected plant in China (https://www.iplant.cn/bhzw/info/782, accessed on 26 February 2026). The increasing pressure on natural resources has created an urgent need for conservation, sustainable utilization, and genetic improvement of this valuable medicinal species.

**Figure 1:**
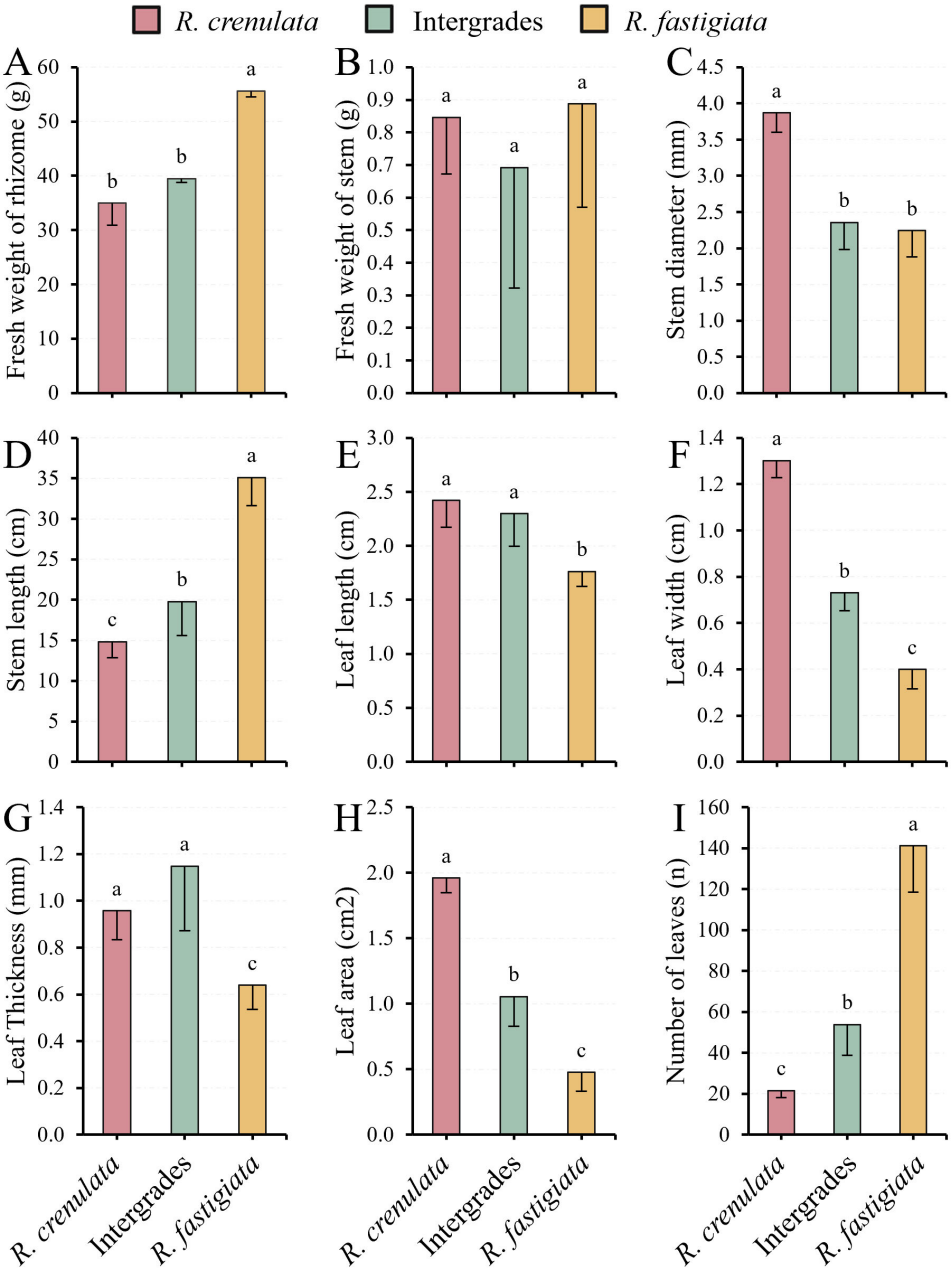
Phenotypic comparison of *R. crenulata*, *R. fastigiata*, and intergrades. **(A)** Fresh rhizome weight per plant; **(B)** Fresh weight of individual stem; **(C)** Stem diameter; **(D)** Stem length; **(E)** Leaf length; **(F)** Leaf width; **(G)** Leaf thickness; **(H)** Leaf area; **(I)** Leaf number per stem. Bars represent the standard error (SE). Different letters represent significant differences (*P* < 0.05).

A major challenge in the utilization of *R. crenulata* is the frequent occurrence of adulteration. Morphologically similar species, such as *R. rosea* and *R. fastigiata*, are commonly marketed as substitutes or adulterants owing to their greater biomass or faster growth (Marchev et al., 2020; Liu et al., 2025). Yet these species differ markedly in phytochemical composition, particularly in the content of salidroside—the principal bioactive glycoside responsible for antioxidant, anti-hypoxic, neuroprotective, and cardioprotective effects (Palmeri et al., 2016; Dong et al., 2023). Salidroside is therefore used as a key quality indicator for *R. crenulata* (Zhu et al., 2025). Notably, the roots and rhizomes of *R. crenulata* contain far higher salidroside levels than its aerial parts (**Figure 1B**), and previous studies have confirmed that salidroside accumulates predominantly in the rhizome (Lan et al., 2013; Zhu et al., 2025). Therefore, accurate authentication and a comprehensive understanding of the molecular basis underlying high salidroside accumulation are essential for both quality control and genetic improvement.

The salidroside biosynthetic pathway has been elucidated in detail through multi-omics integration, revealing a conserved enzymatic cascade (**Figure 1C**). L-tyrosine is converted by 4-hydroxyphenylacetaldehyde synthase (4HPAAS), a bifunctional enzyme that catalyzes sequential decarboxylation and oxidative deamination, to generate 4-hydroxyphenylacetaldehyde (4-HPAA). This aldehyde intermediate is subsequently reduced by 4-hydroxyphenylacetaldehyde reductase (4HPAR) to form tyrosol. In the final step, a UDP-dependent glycosyltransferase (UGT) transfers glucose from UDP-glucose to tyrosol in a regiospecific manner to produce salidroside (Torrens-Spence et al., 2018; Zhang et al., 2024). The upstream tyrosine-derived pathway is widely conserved across plants and microorganisms and can be directly utilized or metabolically engineered for tyrosol production (Schenck and Maeda, 2018; Kanehisa et al., 2023; Liang et al., 2024). In contrast, UGT-catalyzed glycosylation constitutes the decisive regulatory step that determines metabolic flux toward salidroside and its final accumulation (Lan et al., 2013). Differential expression patterns and catalytic efficiencies of UGTs have been implicated in interspecific variation of salidroside content, including differences observed between *R. kirilowii* and *R. chrysanthemifolia* (Zhang et al., 2024). Functional validation further supports the central role of UGTs in salidroside biosynthesis: several UGTs from *R. sachalinensis* catalyze the conversion of tyrosol to salidroside *in vitro* (Yu et al., 2011), and heterologous expression systems have demonstrated that plant-derived UGTs such as *AtUGT85A1* from *Arabidopsis thaliana* (Chung et al., 2017) and *RrUGT33* from *R. rosea* (Torrens-Spence et al., 2018), enable efficient salidroside production in engineered *Escherichia coli*. Nevertheless, endogenous UGTs from *R. crenulata*—the pharmacopoeia-designated source—remain poorly characterized, which limits efforts to enhance medicinal quality through molecular breeding.

Under natural conditions, plants with intermediate morphology (hereafter referred to as “intergrades”) between *R. crenulata* and *R. fastigiata* have been observed in the same cultivated population, exhibiting pronounced phenotypic divergence (**Supplementary Figure 2**). This provides a unique opportunity to explore whether interspecific hybridization could combine the high salidroside content of *R. crenulata* with the high biomass yield of *R. fastigiata*, and to dissect the regulatory mechanisms that separate these traits. However, systematic comparisons of *R. crenulata*, *R. fastigiata*, and the intergrades — particularly regarding morphological, anatomical, metabolic, and transcriptomic differences — remain lacking. Here, we conducted an integrated comparative analysis of phenotypic traits, metabolite profiles, anatomical characteristics, and transcriptomes across *R. crenulata*, *R. fastigiata*, and intergrades. Specifically, we aimed to (i) establish a multi-dimensional framework for authenticating *R. crenulata* and distinguishing it from its common adulterant *R. fastigiata*, (ii) to dissect the regulatory basis of salidroside accumulation, with particular emphasis on species-specific UGTs, and (iii) evaluated the potential of intergrades for genetic improvement of yield and medicinal quality. Our findings provide molecular targets for breeding and support the sustainable utilization of this endangered pharmacopoeia-designated species.

## 2 Materials and methods

### 2.1 Plant material and sampling site

Three-year-old cultivated plants of *Rhodiola crenulata*, *R. fastigiata*, and intergrades were sampled from Bayi District, Nyingchi, Tibet Autonomous Region, China (29.6°N, 91.1°E, 4550 m above sea level), provided by Tibet Rhodiola Pharmaceutical Holding Co.

### 2.2 Phenotypic traits comparison

Fresh plants were immediately subjected to phenotypic characterization, including the number of stems and leaves, as well as leaf area and thickness, rhizome fresh weight, diameter, and length. Leaf area was calculated using ImageJ v1.54. Rhizomes were dissected into apical (Rhizome Apical), middle (Rhizome Middle), and basal (Rhizome Basal) portions, dried at 50 °C for 72 h, ground to a 40-mesh powder, and reserved for subsequent metabolic analysis. For each genotype, three independent biological replicates were evaluated, with each replicate comprising 20 individual plants.

### 2.3 Determination of total flavonoids

Total flavonoid content was determined using the NaNO_2_-Al(NO_3_)_3_-NaOH colorimetric method (Shraim et al., 2021). Dried powder samples (0.5000 g each) from the apical, middle, and basal sections of rhizomes derived from *R. crenulata*, *R. fastigiata*, and intergrades were accurately weighed and extracted with 25 mL of 70% ethanol by ultrasonication at 50 °C for 30 min. After cooling, the extracts were diluted to volume, filtered through a 0.45 μm membrane, and retained as sample solutions. For the standard curve preparation, aliquots (0, 1.0, 2.0, 3.0, 4.0, and 5.0 mL) of rutin standard solution (0.2 mg/mL) were precisely transferred into separate tubes. The colorimetric reaction was performed as follows: each aliquot was supplemented with 60% ethanol to 5.0 mL, followed by sequential addition of 5% NaNO_2_ (0.3 mL, 6 min standing), 10% Al(NO_3_)_3_ (0.3 mL, 6 min standing), and 4% NaOH (4.0 mL). The mixture was then brought to 10 mL with 60% ethanol and allowed to stand for 15 min. Absorbance was measured at 510 nm against a reagent blank, and the calibration curve was constructed. For quantification, 1.0 mL of each sample solution was subjected to the same colorimetric procedure. Total flavonoid content was calculated from the calibration curve and expressed as rutin equivalents (% w/w). Samples from n = 3 independent biological replicates per genotype were analyzed, with sample was measured in triplicate as technical replicates.

### 2.4 Determination of salidroside and tyrosol

Dried powder (0.5000 g; passed through a 20-mesh sieve) of *R. crenulata*, *R. fastigiata*, and intergrades rhizomes was accurately weighed into a stoppered conical flask. Ten milliliters of 70% methanol was precisely added, and the flask was sealed and weighed accurately. Ultrasonic extraction was performed at 30 °C (250 W, 40 kHz) for 30 min. After cooling to room temperature, the flask was reweighed, and the lost weight was compensated with 70% methanol. The mixture was thoroughly shaken, filtered through a 0.45 μm microporous membrane, and the filtrate was used as the sample solution. Chromatographic separation was performed using a Waters E2695–2498 HPLC system (Waters, USA) equipped with a Waters C_18_ column (250 mm × 4.6 mm, 5 μm). The mobile phase consisted of methanol-water (15:85, v/v) under isocratic elution. The column temperature was maintained at 25 °C, the detection wavelength was set at 275 nm, the flow rate was 1.0 mL/min, and the injection volume was 10 μL. Preparation of salidroside and tyrosol standards and the quantitative calculations were performed according to established protocols and pharmacopoeia standards (Chinese Pharmacopoeia, 2025; Torrens-Spence et al., 2018). For each genotype and rhizome position, n = 3 independent biological samples were analyzed, with each sample injected in triplicate.

The HPLC method was validated for linearity, limits of detection (LOD) and quantification (LOQ), precision, and repeatability. Calibration curves were constructed using serially diluted standard solutions, and linearity was evaluated by least-squares regression, yielding R² ≥ 0.9995 for both analytes. LOD and LOQ were calculated according to ICH Q2(R1) guidelines (Cassidy et al., 2025) based on the standard deviation of the response (σ) and the slope (S) of the calibration curve, using the formulas LOD = 3.3σ/S and LOQ = 10σ/S. Intra-day precision was assessed by analyzing the same sample solution six times within a single day, and inter-day precision was evaluated over three consecutive days. Repeatability was determined from six independently prepared sample solutions. Detailed validation parameters for both salidroside and tyrosol are provided in **Supplementary Table 1**.

### 2.5 Anatomical observation

Middle sections of rhizomes from three independent biological plants (n = 3) per genotype were fixed in FAA solution (formaldehyde 4%, glacial acetic acid 5%, ethanol 50%, v/v) at 4 °C for 24 h. After paraffin embedding, 8 μm transverse sections were prepared, stained with safranin and fast green, and mounted with neutral balsam. Images were acquired under a light microscope (SWE-CX63, Servicebio, China) at 200× magnification. For quantitative analysis, five randomly selected fields per sample were analyzed using Aipathwell v2 software (Servicebio, China) to measure periderm thickness, cortex width, and vascular bundle number (Wang et al., 2021).

### 2.6 RNA extraction and library preparation

Fresh rhizome samples of *R. crenulata*, *R. fastigiata*, and intergrades at the same developmental stage were collected. After removing the roots, samples were thoroughly washed, immediately dissected into apical (Rhizome Apical), middle (Rhizome Middle), and basal (Rhizome Basal) segments (1–2 cm), and rapidly frozen in liquid nitrogen. For each genotype, three biological replicates were collected, with each biological replicate consisting of a pooled sample from three individual plants. Total RNA was extracted using TRIzol^®^ Reagent (Invitrogen, USA). Purity assessed using a NanoDrop 2000 (A_260_/A_280_ = 1.9–2.1) and integrity evaluated using an Agilent 2100 Bioanalyzer (RIN ≥ 7.8). Strand-specific libraries were constructed using the NEBNext^®^ Ultra™ RNA Library Prep Kit (NEB, USA). Briefly, mRNA was enriched using oligo(dT) beads, fragmented at 94 °C for 8 min, and converted to double-stranded cDNA. Following end repair, A-tailing, and adapter ligation, libraries were purified with AMPure XP beads and amplified by PCR (15 cycles). After quantification with Qubit 4.0 and quality control on the Agilent 2100 Bioanalyzer, libraries were sequenced on the Illumina NovaSeq 6000 platform (PE150), generating ≥ 6 Gb clean data per sample. Detailed procedures for transcriptome library preparation have been described in our previous study (Mo et al., 2026).

### 2.7 Bioinformatics analysis of transcriptome data

Raw sequencing data were assessed for quality using FastQC v0.11.9 (Wingett and Andrews, 2018), and low-quality reads were filtered using Trimmomatic v0.39 with the following parameters: adapter removal, sliding window trimming (4:20), leading/trailing quality threshold of 20, and minimum read length of 50 bp (Bolger et al., 2014). High-quality clean reads were aligned to the reference genome of *R. crenulata* (https://gigadb.org/dataset/100301) (Fu et al., 2017) using HISAT2 v2.2.1 (Kim et al., 2015) with default parameters and the “--max-intronlen 5000” option, achieving mapping rates > 70%. Gene expression was quantified using featureCounts v2.0.3 with the parameters “-s 2 -p” and normalized to FPKM/TPM (Liao et al., 2014). Differential expression analysis was performed using DESeq2 v1.36.0 with the Wald test, and genes with |log_2_FC| ≥ 1 and adjusted *P*-value < 0.05 were defined as DEGs (Love et al., 2014). Detailed RNA-seq procedures have been described previously (Mo et al., 2026). DEGs were annotated against the NR, Swiss-Prot, KEGG, and COG databases using BLAST+ (Camacho et al., 2009). Gene Ontology (GO) and KEGG pathway enrichment analyses were conducted using clusterProfiler v4.4.0 (Wu et al., 2021).

Single nucleotide polymorphisms (SNPs) were called from the HISAT2-aligned BAM files to assess genome-wide genetic relationships among the three genotypes. Read group information was added to each BAM file using SAMtools v1.17, and variant calling was performed via the BCFtools v1.20 mpileup + call pipeline (--multiallelic-caller --variants-only) (Danecek et al., 2021). Raw SNPs were filtered using BCFtools filter with the following criteria: mapping quality (QUAL) ≥ 30, total read depth (INFO/DP) ≥ 10, minor allele frequency (MAF) ≥ 0.05, and a minimum interval of 5 bp between adjacent SNPs. Filtered SNPs were subjected to principal component analysis (PCA) using PLINK v1.9 with the --pca --allow-extra-chr flags (Chang et al., 2015). The proportion of variance explained by each principal component was calculated from the eigenvalues output by PLINK. The PCA results were visualized using Python v3.8 with the matplotlib library (Hunter, 2007).

### 2.8 Weighted gene co-expression network analysis and hub gene identification

Weighted gene co-expression network analysis (WGCNA) was performed using the WGCNA package v1.72 in R (Langfelder and Horvath, 2008). Low-quality samples and genes were filtered using the goodSamplesGenes() function (minFraction = 0.5) and the genefilter package (var.cutoff = 0.5), respectively. A weighted adjacency matrix was constructed with a soft-thresholding power β selected based on scale-free topology (R² > 0.8). Module eigengenes (MEs) were calculated, and their correlations with phenotypic traits were evaluated using Pearson correlation analysis. Modules significantly associated with target traits (P < 0.001) were identified, and hub genes were selected based on high module membership (kME > 0.8) and intramodular connectivity. Network visualization was conducted using Cytoscape v3.10.0 (Shannon et al., 2003), and top-scoring hub genes were ranked by the maximal clique centrality (MCC) method via the CytoHubba plugin (Chin et al., 2014). A soft-thresholding power of β = 11 was chosen, at which the scale-free topology model fit reached *R*² > 0.8, indicating that the network approximates a scale-free topology. The present dataset, comprising 27 samples, meets the minimum recommendation of 20 samples for robust network construction (Langfelder and Horvath, 2008).

### 2.9 Molecular docking analysis

The three-dimensional structures of candidate UDP-glycosyltransferases (UGTs) were predicted using *AlphaFold3* (Jumper et al., 2021; Abramson et al., 2024), and models with predicted local distance difference test (pLDDT) scores > 70 were retained. The predicted structures were subsequently prepared by removing water molecules and adding polar hydrogen atoms and Kollman charges using AutoDockTools v1.5.7 (Morris et al., 2009), followed by structural visualization and quality assessment using PyMOL (v2.5.4 Schrödinger, LLC). The molecular structure of tyrosol (CAS: 501-94-0; PubChem CID: 10393) was retrieved from the PubChem database (https://pubchem.ncbi.nlm.nih.gov/) in 3D-SDF format. Molecular docking was subsequently performed using AutoDock Vina (Trott and Olson, 2010), with the search space defined by a 60 × 60 × 60 Å grid box centered on the predicted catalytic cavity. Binding affinities (kcal/mol) and interaction modes were analyzed using PyMOL and LigPlot+ (Wallace et al., 1995).

### 2.10 Quantitative Real-Time PCR and statistical analysis

Expression patterns of key genes were validated by quantitative real-time PCR (qRT-PCR) using gene-specific primers (**Supplementary Table 5**). Prior to statistical testing, normality was assessed using the Shapiro-Wilk test, and homogeneity of variances was evaluated using Levene’s test. For phenotypic traits and anatomical measurements, where genotype was the sole factor, statistical significance among groups was determined by one-way ANOVA followed by Tukey’s HSD test for *post-hoc* comparisons. For metabolite data, each genotype–tissue combination was treated as an independent group, and one-way ANOVA with Tukey’s HSD test was applied for pairwise comparisons among all 18 groups. All statistical analyses were performed using R v4.5.1, with P < 0.05 considered statistically significant.

## 3 Results

### 3.1 Phenotypic evaluation

Distinct morphological differences were observed among three-year-old cultivated *R. crenulata*, intergrades and *R. fastigiata* (**Supplementary Figure 2D**). The average rhizome yield of *R. crenulata* was 34.9 g/plant, which did not differ significantly from the intergrades (39.5 g) but was significantly lower than that of *R. fastigiata* (55.6* g*) (P < 0.05) (**Supplementary Table 2**; **Figure 1A**). No statistically significant differences in stem biomass were detected among the three genotypes (**Figure 1B**). Regarding stem morphological characteristics, the stem diameter of *R. crenulata* was significantly greater than that of both intergrades and *R. fastigiata* (**Figure 1C**), whereas its stem length was the shortest, significantly lower than that of the other two genotypes (**Figure 1D**). Leaf morphological analysis revealed that leaf length did not differ significantly between *R. crenulata* and intergrades, but both were significantly greater than that of *R. fastigiata* (**Figure 1E**). Notably, the leaf width of *R. crenulata* reached 1.3 cm, approximately 2-fold that of intergrades and 3-fold that of *R. fastigiata* (**Supplementary Table 2**; **Figure 1F**). Leaf thickness was greatest in intergrades, exceeding both parental lines (**Figure 1G**). The variation in leaf area was consistent with that of leaf width, with *R. crenulata* exhibiting the largest value (**Figure 1H**). The leaf number of *R. crenulata* (22) was significantly lower than that of intergrades (54), amounting to only one-sixth of *R. fastigiata* (141) (**Supplementary Table 2**; **Figure 1I**).

### 3.2 Morpho-anatomical characteristics of rhizome

**Figure 2:**
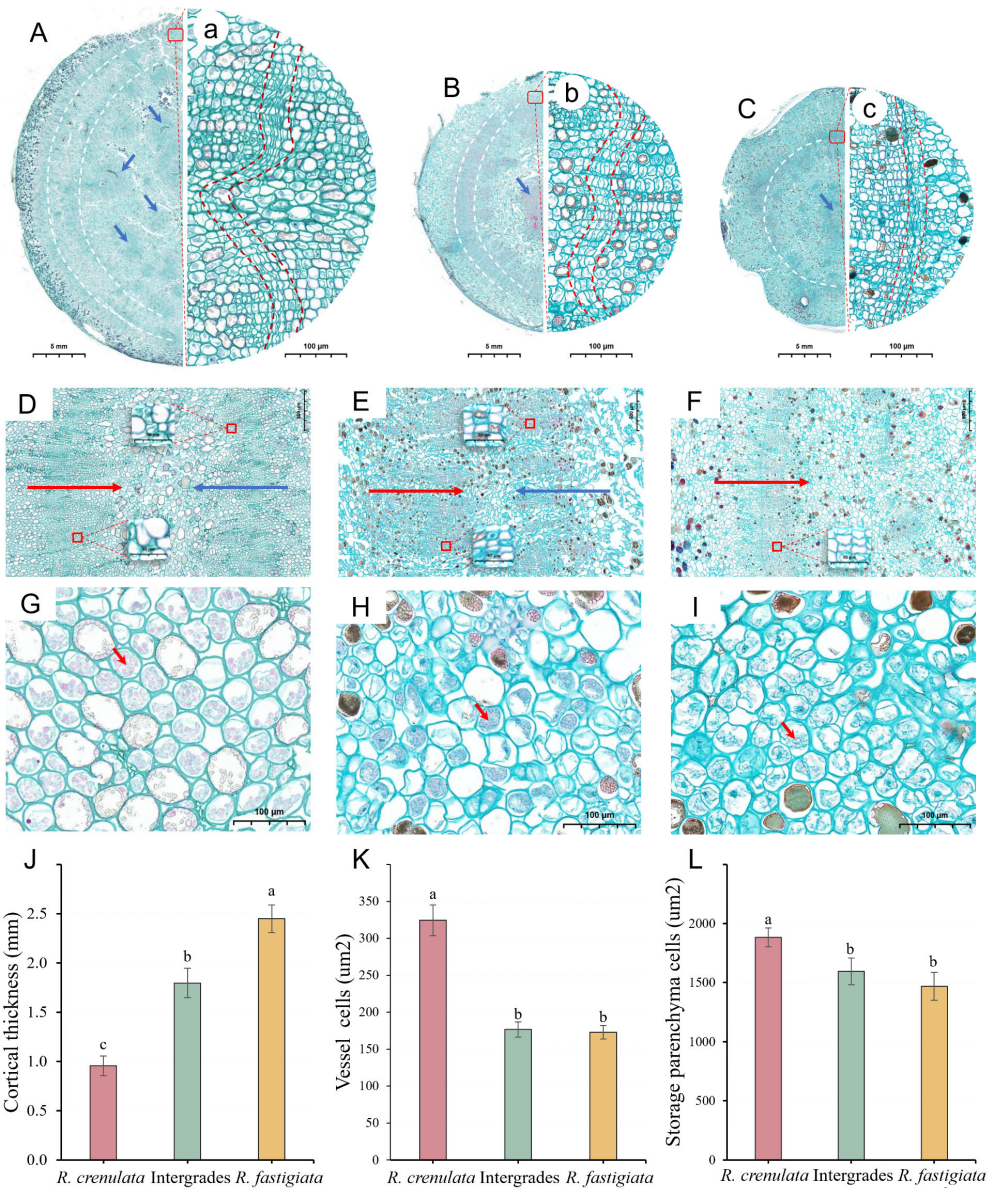
Morpho-anatomical analysis of *R. crenulata*, intergrades and *R. fastigiata*. **(A–C)** Rhizome microstructure and vascular bundles (a–c) of *R. crenulata*, intergrades, and *R. fastigiata*, respectively. White dashed lines indicate vascular bundle cambium; blue arrows point to independent vascular bundles; red dashed lines delimit cambium from the enlarged region marked by red squares. **(D–F)** Cambium structure of vascular bundles in *R. crenulata*, intergrades, and *R. fastigiata*, respectively. Red long arrows indicate xylem development toward the rhizome center; blue long arrows indicate xylem development toward the cortex; enlarged regions marked by red squares show vessel elements of xylem. **(G–I)** Storage parenchyma cells of *R. crenulata*, intergrades and *R. fastigiata*, respectively. Red arrows indicate storage structures and substances within cells. **(J)** Cortex thickness, **(K)** Vessel element size, and **(L)** Storage cell size. Bars represent standard error (SE). Different letters indicate significant differences (P < 0.05).

Paraffin sections of rhizomes stained with safranin-fast green revealed significant differences in microstructure and diameter among *R. crenulata*, intergrades and *R. fastigiata* (**Figure 2**). Specifically, *R. crenulata* exhibited a thicker vascular bundle cambium with two layers of vascular cambium (white dashed circles), multiple independently and randomly distributed vascular bundles (blue arrows), and irregularly arranged cambial meristematic cells (red dashed lines) (**Figures 2A, a**). The intergrades retained the two-layer vascular bundle structure and irregularly arranged cambial meristematic cells of *R. crenulata*, but possessed more numerous and densely arranged independent vascular bundles (**Figures 2B, b**). In contrast, *R. fastigiata* displayed only a single distinct vascular cambium layer with fewer vascular bundles, and its meristematic cells were regularly arranged (**Figures 2C, c**). The rhizome cross-sectional area of *R. crenulata* was significantly larger than that of both intergrades and *R. fastigiata* (**Figure 2D**). Both phloem cells (**Figure 2E**) and xylem cells (**Figure 2F**) of *R. crenulata* were significantly larger than those of intergrades and *R. fastigiata*. Notably, xylem in the enlarged regions (marked by red squares) developed toward the rhizome center (red arrows) (**Figures 2D–F**). However, inward vascular bundle cambium with xylem developing toward the cortex (blue arrows) was observed in both *R. crenulata* and intergrades (**Figures 2D, E**). The interlayer gap between the two cambium layers was wider in *R. crenulata* than in intergrades. Abundant storage parenchyma cells were distributed adjacent to the vascular bundles and in the cortex (**Figures 2G–I**). Further analysis revealed that the cortex thickness of *R. crenulata* was significantly thinner than that of intergrades and *R. fastigiata* (**Figure 2J**), whereas the vessel element area (**Figure 2K**) and storage parenchyma cell area (**Figure 2L**) were significantly larger than those of intergrades and *R. fastigiata*.

### 3.3 Tissue-specific distribution of active compounds

**Figure 3:**
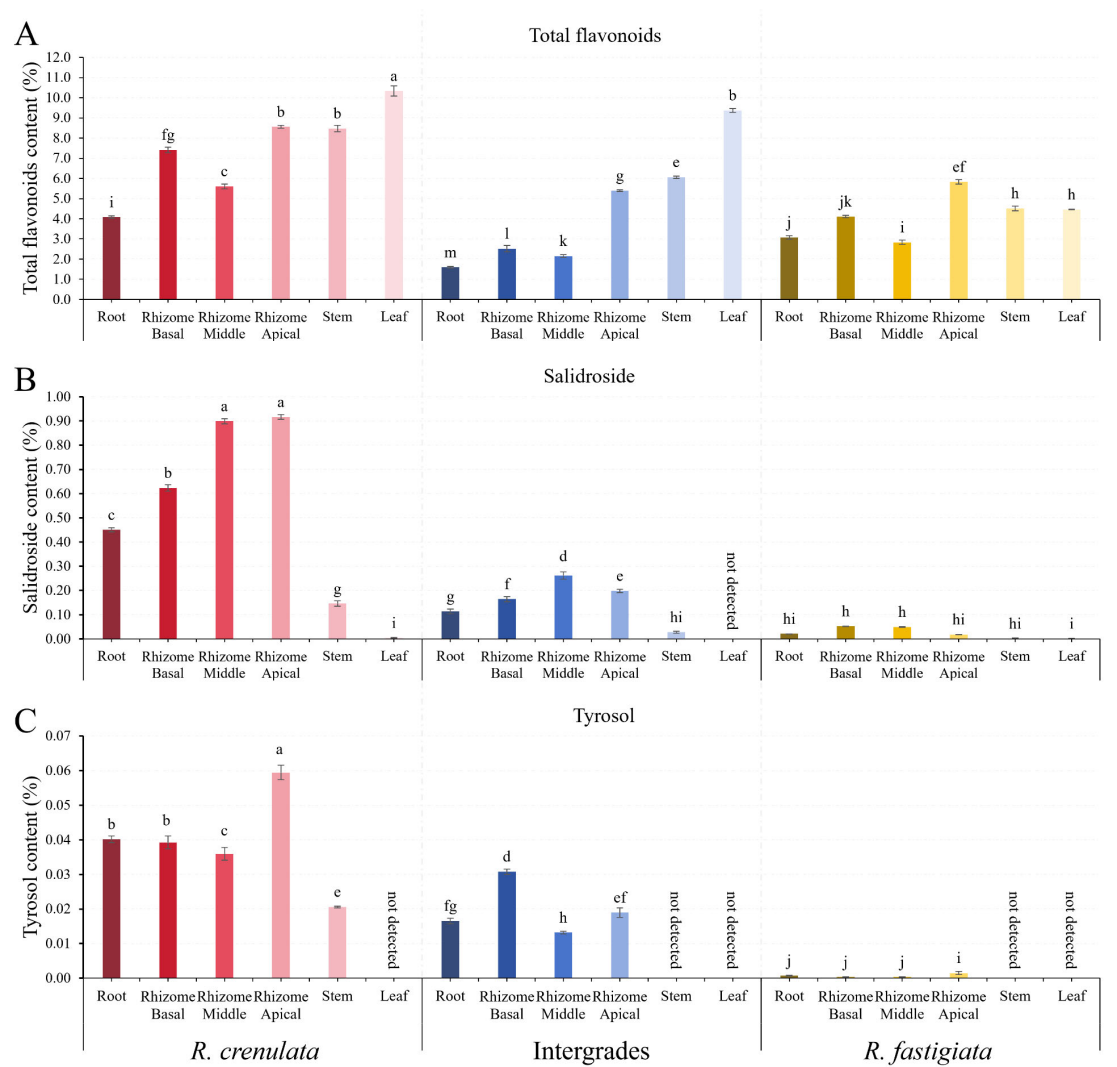
Determination of total flavonoids, salidroside, and tyrosol in *R. crenulata*, intergrades and *R. fastigiata*. **(A)** Total flavonoid content; **(B)** Salidroside content; **(C)** Tyrosol content. Bars represent standard error (SE). Different letters indicate significant differences (P < 0.05).

The tissue-specific distribution of total flavonoids, salidroside, and tyrosol differed markedly among *R. crenulata*, intergrades, and *R. fastigiata* (**Figure 3**). In *R. crenulata*, flavonoids accumulated predominantly in aerial tissues, with the highest levels detected in leaves (10.3%), followed by stems and the rhizome apical (**Figure 3A**; **Supplementary Table 3**). By contrast, salidroside was mainly enriched in rhizome tissues, particularly in the rhizome apical and middle (~0.90%), whereas only trace amounts were detected in stems and the compound was nearly absent in leaves (**Figure 3B**; **Supplementary Table 3**). Tyrosol showed a distinct distribution pattern, reaching its highest levels in roots and rhizomes, with moderate concentrations in stem tissues (**Figure 3C**; **Supplementary Table 3**). A broadly similar pattern was observed in intergrades, although overall metabolite levels were generally lower than those of *R. crenulata*. Specifically, flavonoids were most abundant in leaves and stems, whereas salidroside was primarily confined to rhizome tissues, with the highest accumulation in the rhizome middle (**Figures 3A, B**). Tyrosol occurred at low levels in underground tissues and was undetectable in stems and leaves (**Figure 3C**). In contrast, *R. fastigiata* exhibited markedly lower flavonoid levels and extremely low salidroside and tyrosol contents compared with *R. crenulata* and the intergrades (**Figure 3**). Flavonoids did not exceed 5.8% in the rhizome apical, while salidroside remained below 0.1% in all tissues and tyrosol was nearly undetectable (**Supplementary Table 3**).

### 3.4 Transcriptome profile of rhizome

**Figure 4:**
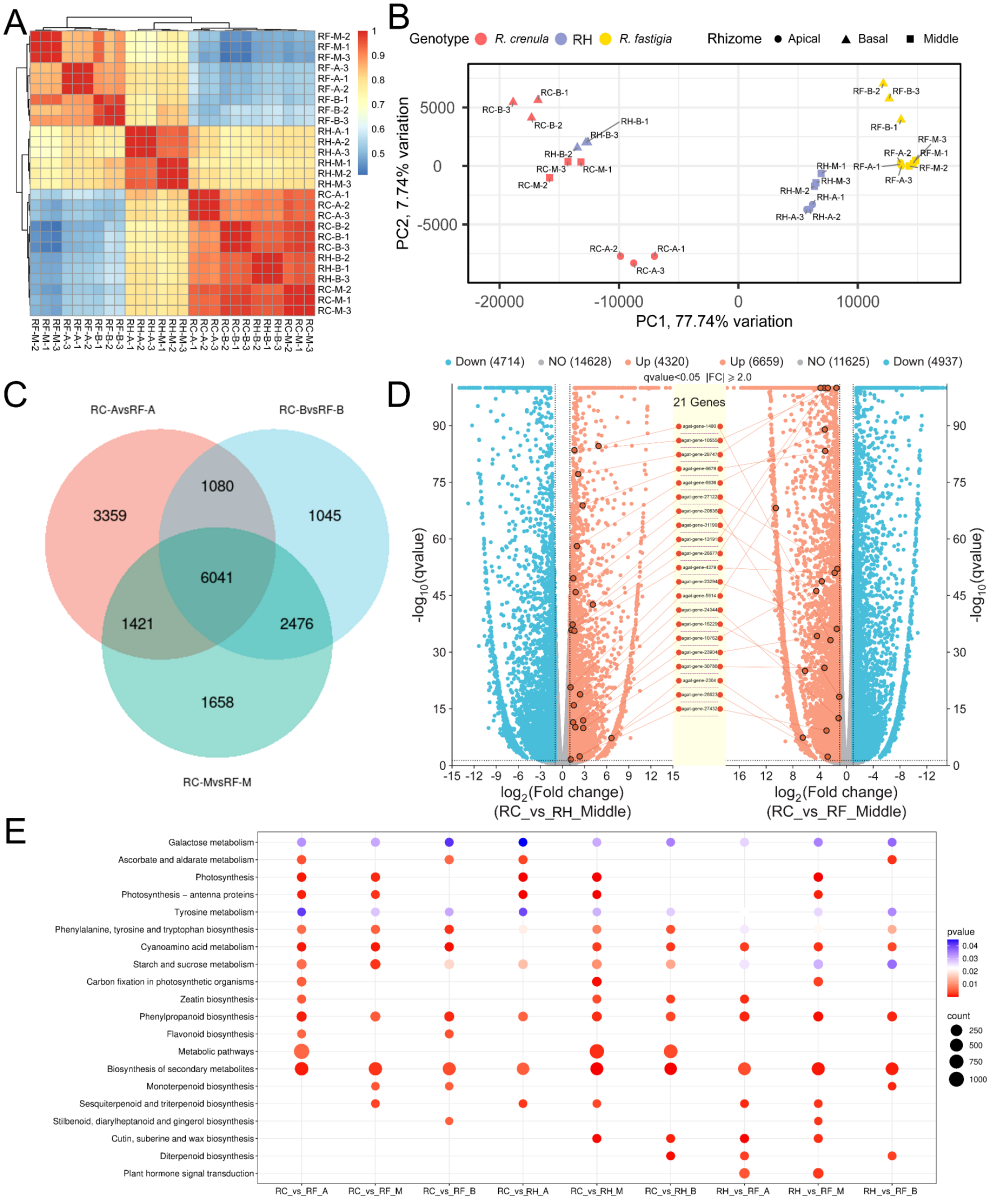
Transcriptome profiles of rhizome tissues in *R. crenulata* (RC), *R. fastigiata* (RF), and intergrades (RH). **(A)** Hierarchical clustering heatmap showing the correlation among samples. **(B)** Principal component analysis (PCA) of transcriptome profiles. **(C)** Venn diagram showing the shared and unique DEGs between *R. crenulata* and *R. fastigiata*. **(D)** Dual volcano plot showing DEGs in the rhizome middle region among *R. crenulata*, intergrades, and *R. fastigiata*. **(E)** KEGG pathway enrichment analysis of DEGs across different pairwise comparisons.

RNA sequencing of 27 biological samples (three genotypes × three rhizome positions × three biological replicates) generated a total of 215.73 Gb of clean bases (**Supplementary Table 4**). All libraries met the required quality thresholds (Q20 > 97%, Q30 > 90%, 45% < GC content < 47%, and clean bases > 6 Gb per sample). Reads showed high mapping efficiency to the reference genome (>72%) and strong reproducibility among biological replicates (r > 0.95) (**Figure 4A**; **Supplementary Table 4**). Principal component analysis (PCA) revealed clear separation among genotypes and rhizome tissues, with PC1 and PC2 explaining 77.74% and 7.74% of the total variance, respectively (**Figure 4B**).

Tissue-specific transcriptomic analysis revealed distinct transcriptional profiles among genotypes and tissue regions (**Figure 4**; **Supplementary Figure 2**). Across the upper (apical), middle, and basal rhizome regions, *R. crenulata* and *R. fastigiata* shared 6, 041 differentially expressed genes (DEGs) (**Figure 4C**), whereas *R. crenulata* and the intergrades shared 2, 684 DEGs (**Supplementary Figure 3A**), and the intergrades and *R. fastigiata* shared 4, 124 DEGs (**Supplementary Figure 3B**). Dual volcano plot analysis further revealed extensive transcriptional divergence among genotypes. In the rhizome middle region, *R. crenulata* vs. intergrades exhibited 9, 034 DEGs, including 4, 320 upregulated and 4, 714 downregulated genes, whereas *R. crenulata* vs. *R. fastigiata* showed 11, 956 DEGs (6, 659 upregulated and 4, 937 downregulated) (**Figure 4D**). In the basal region, 7, 299 DEGs were detected between *R. crenulata* and the intergrades (3, 884 upregulated and 3, 415 downregulated), compared with 10, 642 DEGs between *R. crenulata* and *R. fastigiata* (5, 976 upregulated and 4, 666 downregulated) (**Supplementary Figure 3C**). In the apical region, 8, 405 DEGs were identified between *R. crenulata* and the intergrades (3, 954 upregulated and 4, 551 downregulated), while 11, 901 DEGs were detected between *R. crenulata* and *R. fastigiata* (5, 959 upregulated and 5, 942 downregulated) (**Supplementary Figure 3D**).

KEGG enrichment analysis of DEGs identified 20 significantly enriched metabolic pathways (P < 0.05) across the three genotypes (**Figure 4E**). Among the comparisons, the greatest number of enriched pathways (14) was observed in *R. crenulata* vs. *R. fastigiata* (apical), *R. crenulata* vs. intergrades (middle), and intergrades vs. *R. fastigiata* (middle), whereas the fewest pathways (10) were enriched in the comparison between intergrades and *R. fastigiata* in the basal region. Notably, several metabolic pathways were consistently enriched across all comparisons, including phenylpropanoid biosynthesis (map00940), tyrosine metabolism (map00350), starch and sucrose metabolism (map00500), biosynthesis of secondary metabolites (map01110), galactose metabolism (map00052), and phenylalanine, tyrosine, and tryptophan biosynthesis (map00400).

### 3.5 WGCNA and hub gene identification

**Figure 5:**
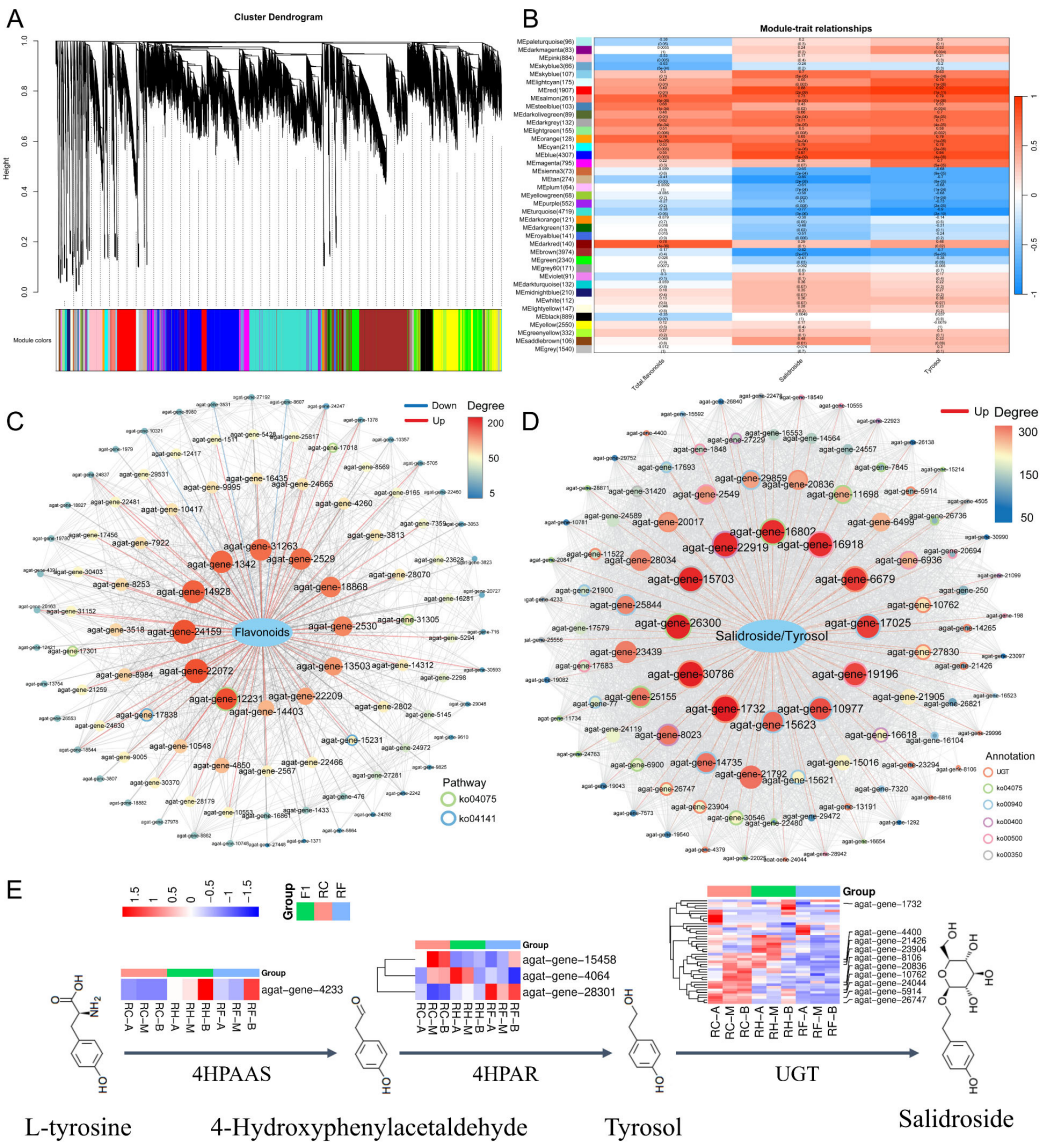
WGCNA analysis and candidate gene screening. **(A)** Gene co-expression module identification. **(B)** Module-metabolite correlation analysis. **(C, D)** Co-expression networks of genes related to specialized metabolite biosynthesis: **(C)** flavonoids, **(D)** salidroside and tyrosol. **(E)** Expression profiles of candidate genes in the salidroside biosynthetic pathway. Network legend: The top 100 genes are presented. Gray lines indicate gene interactions; red and blue lines indicate upregulated and downregulated genes in *R. crenulata* compared with *R. fastigiata*, respectively. Node color intensity (red scale) indicates connectivity degree.

A total of 39 trait-associated modules were identified from 28, 382 expressed genes using weighted gene co-expression network analysis (WGCNA) (**Figures 5A, B**). Total flavonoid content was significantly positively correlated with the MEsalmon module (261 genes), MEorange (128 genes), and MEdarkred (140 genes), with correlation coefficients exceeding 0.74 (*P* < 0.001). Salidroside and tyrosol exhibited similar module association patterns: both traits showed significant positive correlations with the MEsalmon (261 genes), MEdarkgrey (132 genes), and MEcyan (211 genes) (*P* < 0.001, *r* > 0.7). Notably, a strong positive correlation was also observed between the MEred module (1, 907 genes) and the MEblue (4, 307 genes) (*P* < 0.001, *r* > 0.84).

In the flavonoid-associated co-expression network (**Figure 5C**), the top 100 genes ranked by degree centrality were primarily enriched in the pathways of plant hormone signal transduction (ko04075) and protein processing in the endoplasmic reticulum (ko04141). For instance, the gene with the highest degree value, agat-gene-12231, was annotated as K14484, which is associated with indole-3-acetic acid (IAA) signaling. In the co-expression network associated with salidroside and tyrosol (**Figure 5D**), the top 100 hub genes were significantly enriched in multiple metabolic pathways, including tyrosine metabolism (ko00350), phenylalanine, tyrosine, and tryptophan biosynthesis (ko00400), starch and sucrose metabolism (ko00500), phenylpropanoid biosynthesis (ko00940), and plant hormone signal transduction (ko04075). In addition, a total of 21 genes were annotated as UDP-glycosyltransferases (UGTs).

Further analysis of the salidroside biosynthesis pathway (**Figure 5E**) identified 45 key differentially expressed genes. These included one *Rc4HPAAS* gene (*agat-gene-4233*), three *Rc4HPAR* genes (*agat-gene-15458*, *agat-gene-4064*, and *agat-gene-28301*), and 42 differentially expressed *RcUGT* genes.

### 3.6 Identification and functional characterization of key RcUGTs

**Figure 6:**
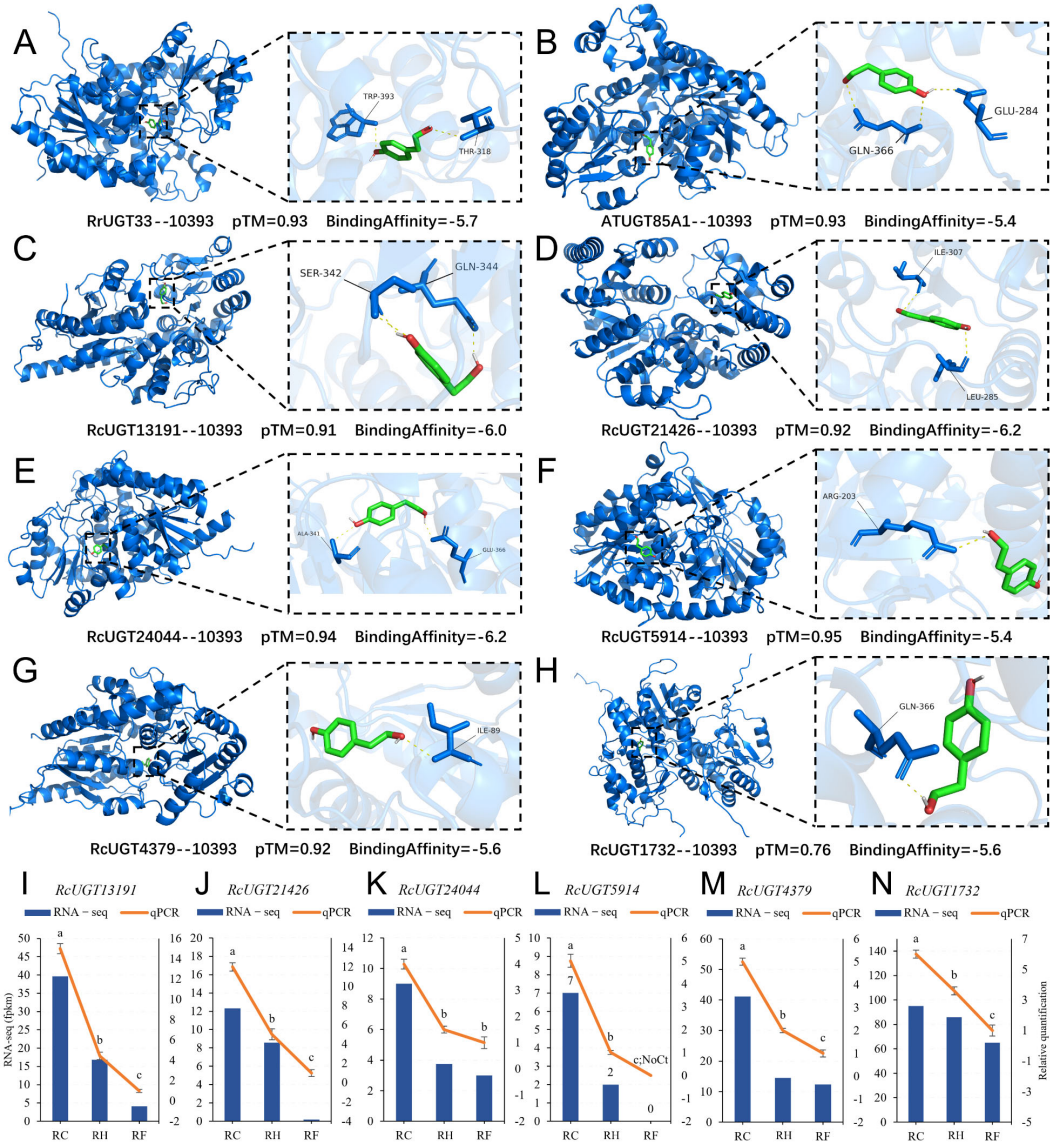
Molecular docking of UGT proteins with tyrosol and expression validation. **(A, B)** Docking poses of functionally characterized positive controls RrUGT33 and AtUGT85A1 with tyrosol (PubChem CID: 10393). **(C–H)** Molecular docking conformations of candidate *Rc*UGT genes with tyrosol. **(I–N)** qPCR expression profiling of candidate *Rc*UGT genes. Legend: **(A–H)**, pTM (predicted TM-score) indicates AlphaFold3 structure prediction confidence. Magnified views (dashed boxes) display hydrogen bonding interactions (yellow dashed lines) between amino acid residues and tyrosol.

Molecular docking analysis using tyrosol (PubChem CID: 10393) as the ligand was first performed with the functionally validated enzyme RrUGT33 as a positive control. Tyrosol could be stably accommodated within the binding pocket of RrUGT33 (pTM = 0.93), with a binding energy of −5.7 kcal/mol (**Figure 6A**). Structural inspection further revealed that the residues TRP-393 and THR-318 formed stable hydrogen bonds with the terminal hydroxyl groups of the ligand, as indicated by yellow dashed lines. Similarly, tyrosol exhibited stable binding with the experimentally validated enzyme AtUGT85A1 (pTM = 0.93), with a binding energy of −5.4 kcal/mol. Hydrogen bond interactions were observed between the ligand and residues GLU-284 and GLN-366 (**Figure 6B**).

A total of 10 differentially expressed genes that were specifically and highly expressed in *R. crenulata* were identified within the salidroside biosynthesis pathway (**Figure 5E**). Based on molecular docking analysis, six RcUGT genes were selected as candidate genes, including *RcUGT13191* (*agat-gene-13191*), *RcUGT21426* (*agat-gene-21426*), *RcUGT24044* (*agat-gene-24044*), *RcUGT5914* (*agat-gene-5914*), *RcUGT4379* (*agat-gene-4379*), and *RcUGT1732* (*agat-gene-1732*). All candidates exhibited binding energies below −5.4 kcal/mol, indicating strong substrate-binding potential. Among them, *RcUGT13191*, *RcUGT21426*, and *RcUGT24044* showed binding energies lower than −6.0 kcal/mol, suggesting higher catalytic potential.

The expression patterns of these six candidate genes were validated by qRT-PCR, confirming their high expression levels in *R. crenulata* (**Figures 6I–N**). Notably, *RcUGT5914* was not expressed in *R. fastigiata* (**Figure 6L**), identifying it as a species-specific candidate potentially involved in salidroside biosynthesis.

## 4 Discussion

As an endangered species designated as the sole medicinal source in the Chinese Pharmacopoeia, *Rhodiola crenulata* faces multiple challenges, including overexploitation, habitat destruction, and genetic resource loss (Zhu et al., 2025). The intergrades obtained under natural conditions exhibited pronounced phenotypic divergence (**Supplementary Figure 2D**), suggestive of introgression or distant hybridization events. At the transcriptome-wide SNP level, PC1 and PC2 together explained 78.8% of the total genetic variance, and the intergrades samples occupied an intermediate genetic position between *R. crenulata* and *R. fastigiata* — a genetic profile characteristic of hybrid intermediacy (**Supplementary Figure 4**). This further underscore the urgency of germplasm conservation and sustainable utilization. This research, phenotypic, anatomical, metabolomic, and transcriptomic differences among *R. crenulata*, *R. fastigiata*, and their intergrades were systematically compared, providing new insights into species discrimination and the molecular mechanisms underlying medicinal quality.

Domestication and cultivation represent a critical pathway to sustainable utilization. Wild *R. crenulata* requires several years to decades to accumulate sufficient biomass (**Supplementary Figure 1A**), making artificial domestication and cultivation essential. In this study, cultivated *R. crenulata* materials met the quality standards of Chinese Pharmacopoeia (**Figure 3**; **Supplementary Figure 2**), confirming their feasibility as medicinal materials. Domestication of medicinal plants is often accompanied by a “domestication syndrome, ” involving coordinated changes in morphology, metabolism, and reproduction. For instance, cultivated *Agastache mexicana* exhibits enlarged reproductive organs, enhanced vegetative propagation, and increased accumulation of secondary metabolite (Carrillo-Galván et al., 2020). Similarly, cultivated *Lonicera japonica* shows improved floral traits, yield, and bioactive compound content (Hou et al., 2024). Meanwhile, overharvesting has led to severe depletion of wild *Scrophularia ningpoensis*, making cultivation an essential alternative (Chen et al., 2014). In *Atractylodes macrocephala*, domestication-associated shifts in genetic diversity further highlight the importance of maintaining gene flow between wild and cultivated populations (Chen et al., 2019). Collectively, domestication represents not only a shift in production mode but also a coordinated process involving resource sustainability, quality improvement, and biodiversity conservation.

Accurate authentication of *R. crenulata* is a prerequisite for ensuring its medicinal quality and clinical safety. Our integrated analysis of morphological, anatomical, metabolic, and transcriptomic features reliably distinguishes *R. crenulata* from *R. fastigiata* and intergrades (**Figures 1**–Belintani et al., 2023). Because medicinal materials are often traded in dried or powdered forms, traditional morphological identification is frequently constrained, leading to widespread adulteration and misidentification (Sánchez et al., 2020). Market surveys have indicated that authentic *R. crenulata* materials account for less than 40% of commercial samples, with substitution by at least 20 other species (Xin et al., 2015; Cunningham et al., 2020), underscoring the limitations of single-method identification. Although DNA barcoding provides an effective tool for species authentication in *Rhodiola*, its resolution remains insufficient for differentiating closely related taxa, necessitating integrative approaches (Zhang et al., 2015). Transcriptome-wide comparisons have proven effective for species delimitation in diverse taxa, such as triatomine insects and ciliates, by leveraging differentially expressed genes and orthologous variation (Belintani et al., 2023; Shazib et al., 2025). Therefore, our multi-omics framework enables more accurate discrimination of *R. crenulata* and provides critical insights into the molecular basis underlying variation in bioactive compounds.

Differential activity of endogenous UGT enzymes likely underlies the superior salidroside accumulation observed in *R. crenulata* compared with other *Rhodiola* species. The universally low tyrosol levels (< 0.06%; **Figure 3**) across *Rhodiola* species indicate that differential UGT conversion efficiency may serve as the primary constraint on salidroside accumulation (Kotsupiy et al., 2024). Functional enrichment of hub genes further indicated that these genes are mainly involved in plant hormone signaling, amino acid metabolism, and phenylpropanoid biosynthesis pathways (**Figures 3**, **5**), suggesting that both precursor supply and downstream modifications collectively shape metabolite accumulation (Ahmad et al., 2025). UDP-glycosyltransferases (UGTs) play a central role in salidroside biosynthesis by catalyzing the glycosylation of tyrosol, a key step determining salidroside formation (Palmeri et al., 2016; Schenck and Maeda, 2018; Liang et al., 2024). Several UGTs involved in this pathway have been functionally characterized in non-*R. crenulata* species. For instance, *RsUGT73B6* was the first identified glucosyltransferase participating in salidroside biosynthesis (Ma et al., 2007), whereas *RsUGT72B14* exhibited substantially higher catalytic efficiency than *RsUGT74R1* (Yu et al., 2011). In addition, *RrUGT33* showed the highest catalytic activity toward tyrosol among *RrUGTs* (Torrens-Spence et al., 2018). However, RcUGTs derived from *R. crenulata*, the pharmacopoeia-designated authentic source species, remain largely unexplored. In this study, by integrating co-expression analysis, molecular docking, and expression validation, six RcUGTs were identified as high-confidence candidates (**Figures 6C–I**). Notably, *RcUGT5914* displayed species-specific expression and was absent in *R. fastigiata*, suggesting a potential role in species-dependent metabolite differentiation.

This study also provides important implications for the utilization and improvement of *R. crenulata*. Unlike previous reports in medicinal plants such as *Bupleurum chinense* (Mo et al., 2026) and *Eucommia ulmoides* (Li et al., 2019), where F_1_ hybrids often exhibit pronounced heterosis. The intergrades in this study did not show significant advantages in yield or metabolite accumulation (**Figure 1A**; **Figure 3B**). This suggests that, at least under the conditions tested and pending further genetic confirmation of the intergrades, *R. fastigiata* may be a suboptimal parental donor for improving *R. crenulata*.

Several limitations should be acknowledged. All *Rhodiola* species are currently listed as nationally protected plants in China, and the cultivation-based domestication of *R. crenulata* remains at an early exploratory stage; consequently, multi-site or multi-year cultivated populations are not yet available. Against this backdrop, the present study represents an early effort to characterize quality traits of cultivated *R. crenulata* alongside its adulterant and intergrades under controlled conditions. First, all samples were collected from a single cultivation site at a single harvest time, which controls for environmental variation but limits generalizability across locations and growing seasons. Second, the intergrades identity, while supported by SNP-based PCA, warrants further genetic confirmation. Third, the candidate UGTs identified through in silico screening require functional validation through heterologous expression and *in vitro* enzyme assays. Fourth, although the 27 samples used for WGCNA satisfy the recommended minimum of 20 samples (Langfelder and Horvath, 2008) and a stringent soft-thresholding power was applied to minimize spurious correlations, validation of key hub genes in larger independent datasets would further strengthen these findings. Despite these caveats, the global market for *R. crenulata* was valued at $178 million in 2024 and is forecast to reach $412 million by 2033, with a compound annual growth rate (CAGR) of 9.7%, indicating substantial economic potential (Anuradha B. More, 2025). Therefore, improving cultivated *R. crenulata* through molecular breeding remains a priority. Our identification of *RcUGT5914* and other candidate UGTs provides direct targets for functional validation and for marker-assisted selection. Future efforts should focus on breaking the negative correlation between biomass accumulation and salidroside content by engineering regulatory networks, and on developing molecular breeding strategies to enhance both yield and medicinal quality, thereby supporting the sustainable utilization of this pharmacopoeia-designated species.

## 5 Conclusion

This study systematically compared *R. crenulata*, *R. fastigiata*, and intergrades using an integrative multi-dimensional framework encompassing morphology, anatomy, metabolite profiling, and transcriptomics. Distinct differences across all four dimensions enabled reliable species discrimination and clarified key traits associated with medicinal quality. We identified six candidate UGT genes, particularly the species-specific *RcUGT5914*, providing promising targets for future functional validation, molecular breeding, and metabolic engineering. The absence of yield heterosis in the intergrades suggests that *R. fastigiata* is not an ideal parental donor for improving *R. crenulata*. Collectively, this work advances the accurate authentication and quality evaluation of *R. crenulata* and provides a molecular foundation for the genetic improvement and sustainable utilization.

## Data Availability

The RNA-seq data that support the findings of this study have been deposited into CNSA with accession number CNP0009380.
