# Supplementary material for: Quality differentiation and salidroside biosynthesis in Rhodiola crenulata, R. fastigiata, and intergrades
Source: Front Plant Sci. 2026 Jun 29;17:1873808. doi: 10.3389/fpls.2026.1873808 (PMC13357708; doi:10.3389/fpls.2026.1873808)
Supplement: Supplementary file 1 [file DataSheet1.docx]

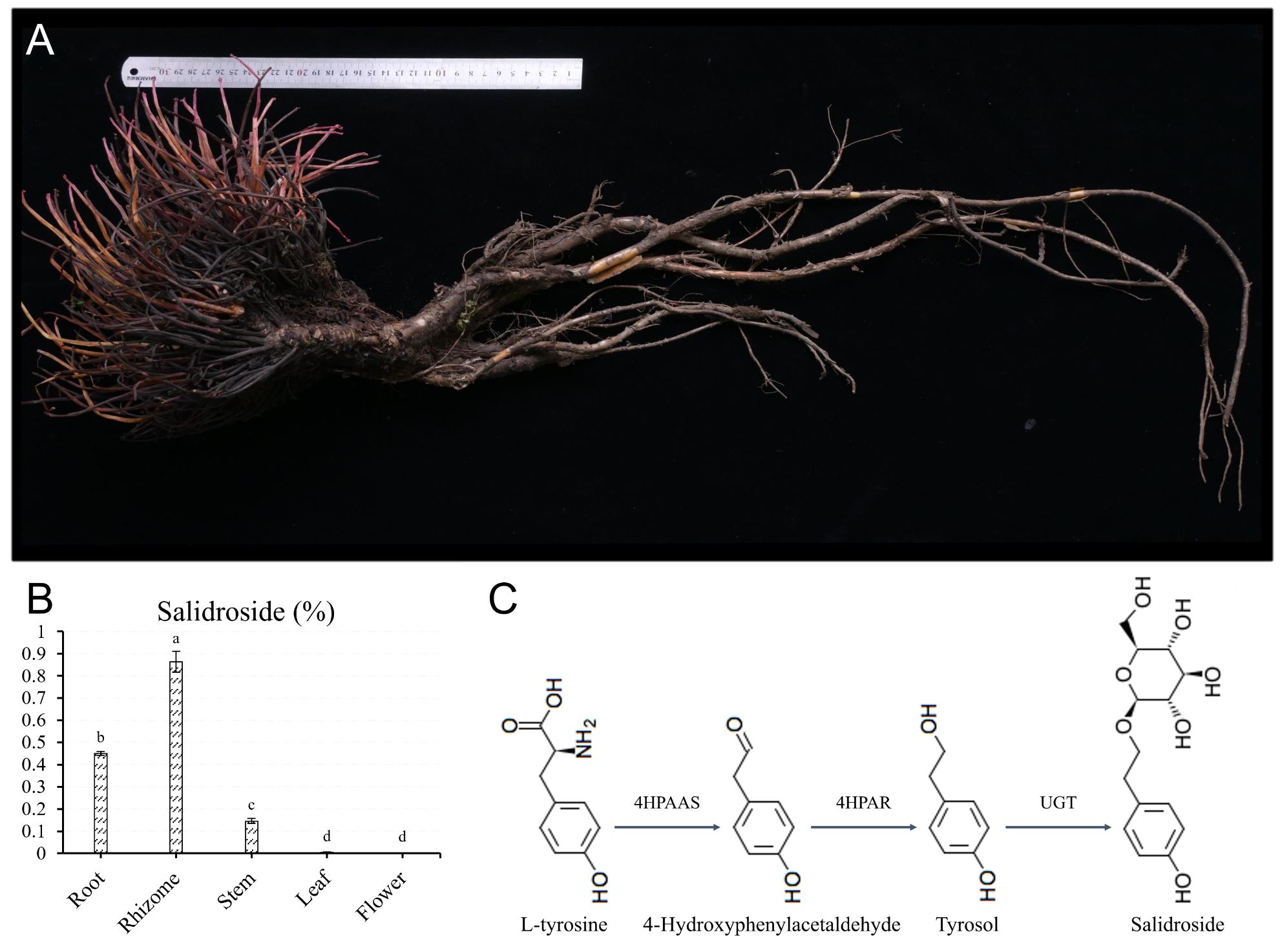


Figure S1 Morphology, salidroside content, and biosynthetic pathway of *R. crenulata*. (A) Wild R. crenulata plants over 30 years old. (B) Tissue-specific distribution of salidroside content in *R. crenulata*. (C) Proposed simplified biosynthetic pathway of salidroside.


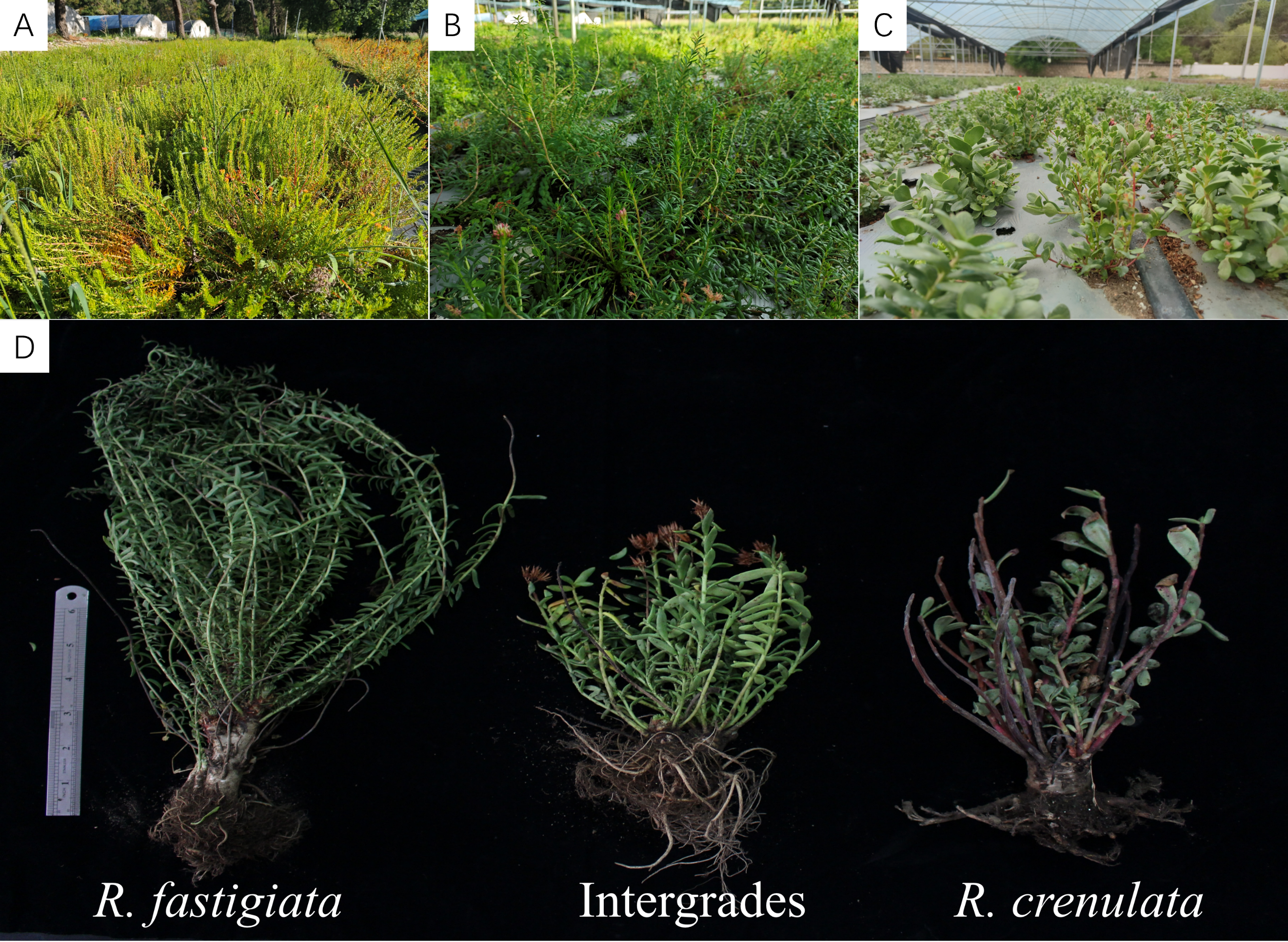


Figure S2 Morphology of cultivated *R. crenulata*, intergrades, and *R. fastigiata*. (A–C) Field performance of *R. fastigiata* (A), intergrades (B), and *R. crenulata* (C). (D) Whole-plant morphology of three-year-old individual specimens.


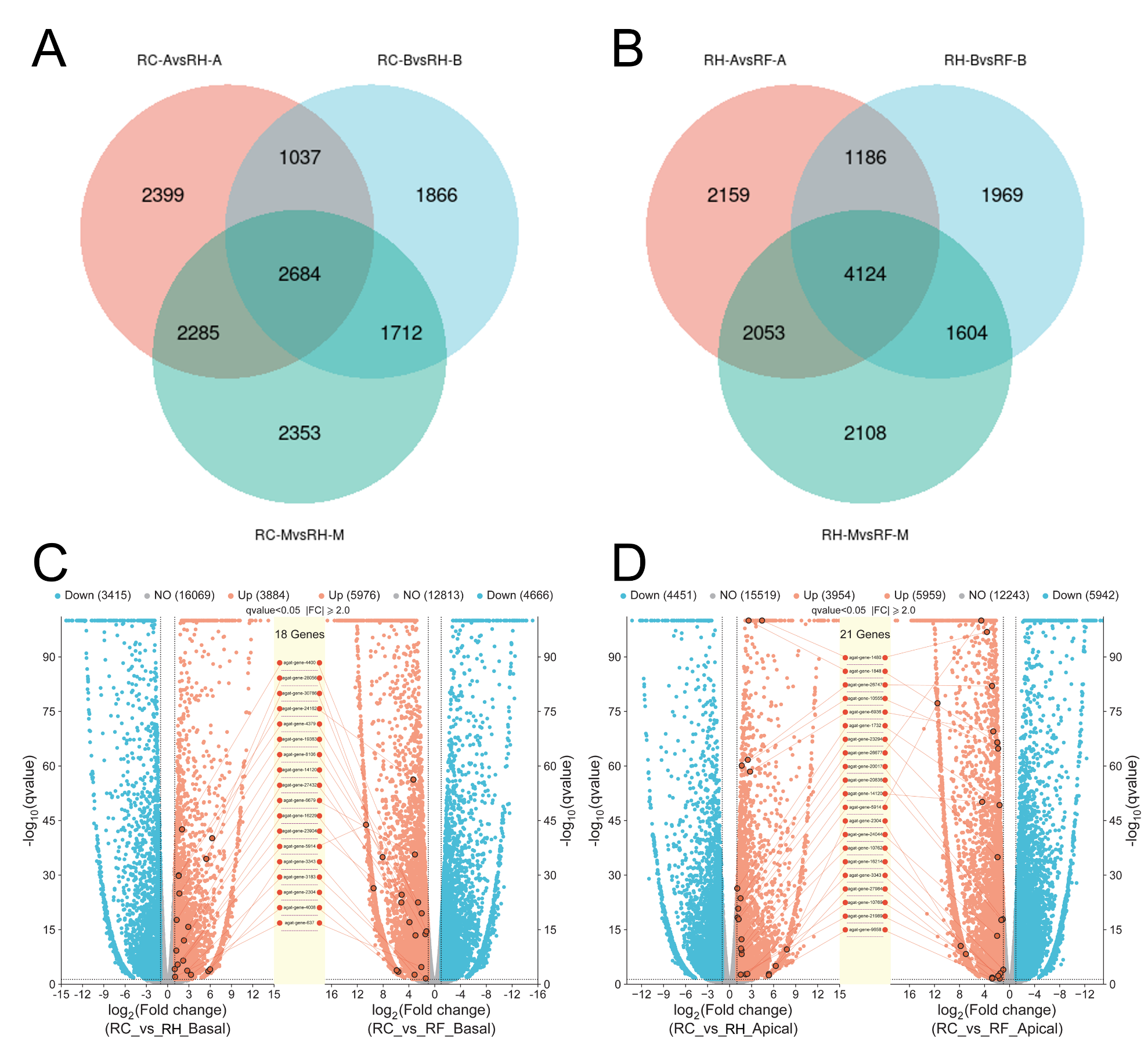


Figure S3 Transcriptome profiles of rhizome tissues in *R. crenulata* (RC), *R. fastigiata* (RF), and intergrades (RH). (A) Venn diagram of differentially expressed genes (DEGs) between *R. crenulata* and intergrades. (B) Venn diagram of DEGs between intergrades and *R. fastigiata*. (C) Dual volcano plots of DEGs in the basal rhizome region for *R. crenulata* vs. intergrades and *R. crenulata* vs. *R. fastigiata*. (D) Dual volcano plots of DEGs in the apical rhizome region for *R. crenulata* vs. intergrades and *R. crenulata* vs. *R. fastigiata*.


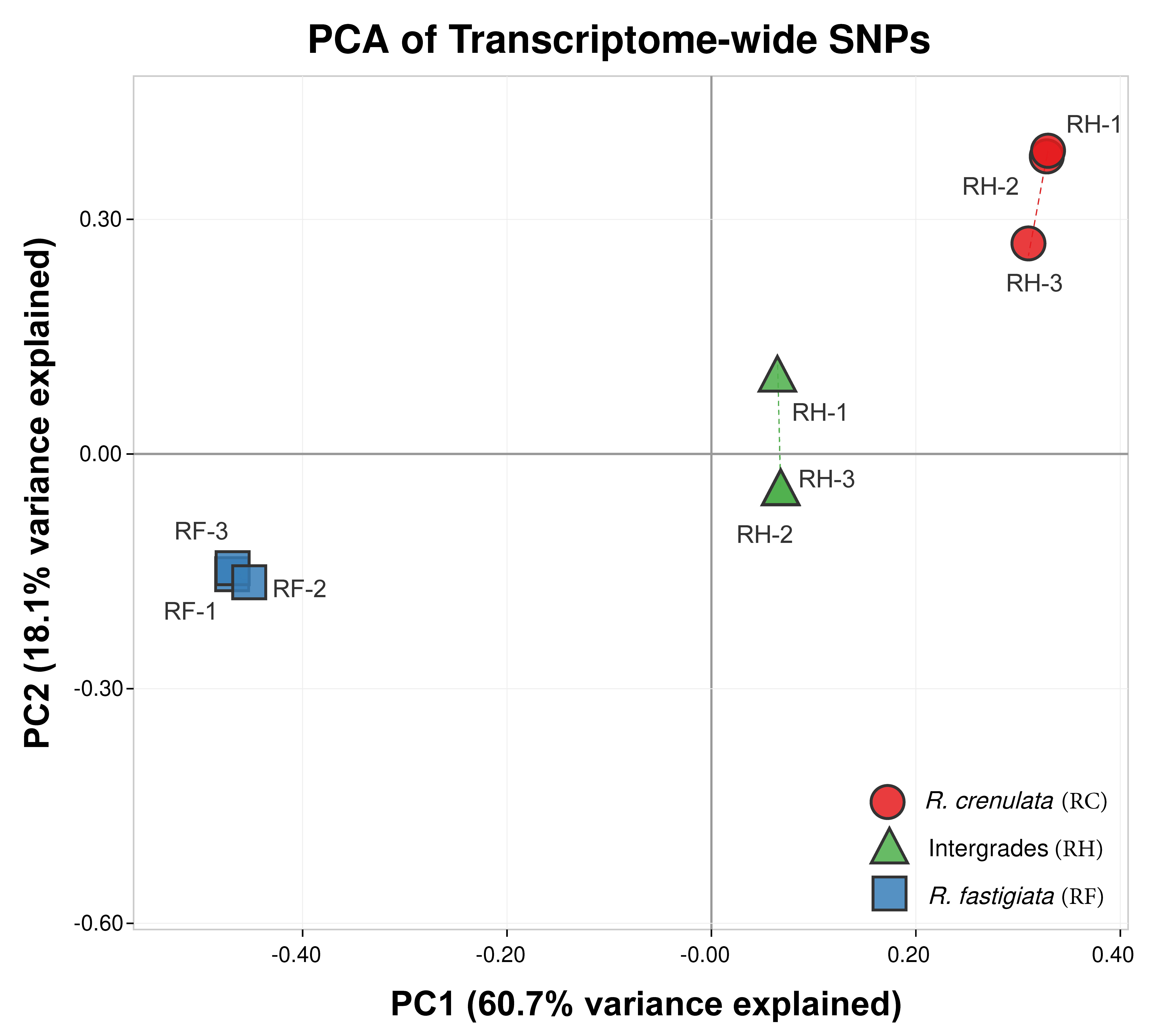


Figure S4 Transcriptome-based SNP analysis of the *R. crenulate* (RC), intergrades (RH), and *R. fastigiata* (RF). **−1**, rhizome apical; **−2**, rhizome middle; **−3**, rhizome basal.
