# Supplementary material for: Quality differentiation and salidroside biosynthesis in Rhodiola crenulata, R. fastigiata, and intergrades
Source: Front Plant Sci. 2026 Jun 29;17:1873808. doi: 10.3389/fpls.2026.1873808 (PMC13357708; doi:10.3389/fpls.2026.1873808)
Supplement: Supplementary file 2 [file DataSheet2.docx]

Table S1 Method validation parameters for the HPLC determination.

| Parameter | Salidroside | Tyrosol |
| --- | --- | --- |
| Retention time (min) | 8.1 | 10.2 |
| Calibration equation | Y = 0.02X − 20 | Y = 0.008X − 10 |
| R^2^ | 0.9995 | 0.9997 |
| Linear range (μg/mL) | 50 – 500 | 50 – 1000 |
| LOD (μg/mL) | 1.01 | 1.13 |
| LOQ (μg/mL) | 4.12 | 4.57 |
| Intra-day precision, RSD (%) | 0.86 | 0.81 |
| Inter-day precision, RSD (%) | 0.98 | 0.86 |
| Repeatability, RSD (%) | 1.61 | 1.47 |

Note. Y = concentration (μg/mL); X = peak area (μV·s).

Table S2 Phenotypic comparison of *R. crenulata*, *R. fastigiata,* and intergrades materials.

| Phenotype | *R. crenulata* | | | intergrades | | | *R. fastigiata* | | |
| --- | --- | --- | --- | --- | --- | --- | --- | --- | --- |
|  | Mean | SE | sig | Mean | SE | sig | Mean | SE | sig |
| Fresh weight of rhizome (g) | 34.9 | 4.02 | b | 39.5 | 0.69 | b | 55.6 | 1.08 | a |
| Fresh weight of stem (g) | 0.8 | 0.17 | a | 0.7 | 0.37 | a | 0.9 | 0.32 | a |
| Stem diameter (mm) | 3.9 | 0.27 | a | 2.4 | 0.37 | b | 2.2 | 0.37 | b |
| Stem length (cm) | 14.8 | 1.93 | c | 19.8 | 4.21 | b | 35.1 | 3.44 | a |
| Leaf length (cm) | 2.4 | 0.25 | a | 2.3 | 0.30 | a | 1.8 | 0.14 | b |
| Leaf width (cm) | 1.3 | 0.07 | a | 0.7 | 0.08 | b | 0.4 | 0.08 | c |
| Leaf Thickness (mm) | 1.0 | 0.12 | a | 1.1 | 0.28 | a | 0.6 | 0.10 | c |
| Leaf area (cm2) | 2.0 | 0.11 | a | 1.1 | 0.23 | b | 0.5 | 0.15 | c |
| Leaf perimeter (cm) | 6.1 | 0.54 | a | 5.1 | 0.65 | b | 3.9 | 0.59 | c |
| Number of leaves (n) | 21.6 | 3.47 | c | 53.8 | 14.94 | b | 141.2 | 22.56 | a |

Table S3 Metabolic analysis of *R. crenulata*, *R. fastigiata,* and intergrades materials.

| Genotype | Tissue | Total flavonoids (%) | | | Salidroside (%) | | | Tyrosol (‰) | | |
| --- | --- | --- | --- | --- | --- | --- | --- | --- | --- | --- |
|  |  | Mean | SE | sig. | Mean | SE | sig. | Mean | SE | sig. |
| *R. crenulata* | Root | 4.084 | 0.06 | i | 0.4506 | 0.0084 | c | 0.401 | 0.010 | b |
|  | Rhizome Basal | 7.412 | 0.13 | fg | 0.6231 | 0.0132 | b | 0.392 | 0.018 | b |
|  | Rhizome Middle | 5.600 | 0.12 | c | 0.8991 | 0.0105 | a | 0.359 | 0.018 | c |
|  | Rhizome Apical | 8.556 | 0.07 | b | 0.9164 | 0.0095 | a | 0.594 | 0.021 | a |
|  | Stem | 8.472 | 0.16 | b | 0.1457 | 0.0113 | g | 0.206 | 0.002 | e |
|  | Leaf | 10.334 | 0.26 | a | 0.0041 | 0.0023 | **i** | 0.000 | 0.000 | NA |
| Intergrades | Root | 1.596 | 0.04 | m | 0.1139 | 0.0098 | g | 0.165 | 0.008 | fg |
|  | Rhizome Basal | 2.518 | 0.16 | l | 0.1642 | 0.0103 | f | 0.308 | 0.008 | d |
|  | Rhizome Middle | 2.149 | 0.07 | k | 0.2621 | 0.0148 | d | 0.132 | 0.004 | h |
|  | Rhizome Apical | 5.397 | 0.04 | g | 0.1986 | 0.0067 | e | 0.190 | 0.014 | ef |
|  | Stem | 6.059 | 0.05 | e | 0.0276 | 0.0036 | hi | 0.000 | 0.000 | NA |
|  | Leaf | 9.369 | 0.09 | b | 0.0000 | 0.0000 | NA | 0.000 | 0.000 | NA |
| *R. fastigiata* | Root | 3.078 | 0.09 | j | 0.0210 | 0.0002 | hi | 0.007 | 0.001 | j |
|  | Rhizome Basal | 4.106 | 0.06 | jk | 0.0527 | 0.0001 | h | 0.003 | 0.001 | j |
|  | Rhizome Middle | 2.831 | 0.11 | i | 0.0493 | 0.0024 | h | 0.004 | 0.001 | j |
|  | Rhizome Apical | 5.833 | 0.11 | ef | 0.0175 | 0.0007 | hi | 0.015 | 0.005 | i |
|  | Stem | 4.512 | 0.12 | h | 0.0014 | 0.0023 | hi | 0.000 | 0.000 | NA |
|  | Leaf | 4.456 | 0.02 | h | 0.0014 | 0.0008 | i | 0.000 | 0.000 | NA |

Note. NA means not detected.

Table S4 Transcriptome data statistics

| genotype | rhizome | SampleID | Clean Bases (Gb) | Clean GC (%) | Clean Q20 (%) | Clean Q30 (%) | Total mapped (%) |
| --- | --- | --- | --- | --- | --- | --- | --- |
| *R. crenulata* | Apical | RC-A-1 | 8.97 | 45.98;45.97 | 98.23;97.87 | 91.41;90.56 | 88.79 |
|  |  | RC-A-2 | 6.64 | 45.85;45.87 | 98.16;97.75 | 91.16;90.15 | 91.03 |
|  |  | RC-A-3 | 7.66 | 45.93;45.94 | 98.19;97.92 | 91.31;90.78 | 90.70 |
|  | Middle | RC-M-1 | 7.23 | 45.93;45.96 | 98.12;97.73 | 90.95;89.99 | 90.80 |
|  |  | RC-M-2 | 8.61 | 46.15;46.16 | 98.00;97.83 | 90.58;90.35 | 90.38 |
|  |  | RC-M-3 | 9.90 | 45.91;45.97 | 98.54;98.11 | 92.70;91.61 | 91.24 |
|  | Basal | RC-B-1 | 7.81 | 45.97;45.98 | 98.00;97.84 | 90.46;90.33 | 90.89 |
|  |  | RC-B-2 | 7.29 | 45.87;45.89 | 98.25;97.95 | 91.31;90.62 | 91.09 |
|  |  | RC-B-3 | 7.63 | 45.99;46.00 | 98.15;97.75 | 91.13;90.14 | 90.34 |
| intergrades | Apical | RH-A-1 | 10.53 | 45.64;45.64 | 98.52;98.39 | 92.38;92.38 | 82.99 |
|  |  | RH-A-2 | 6.73 | 45.77;45.78 | 98.09;97.82 | 90.82;90.27 | 81.91 |
|  |  | RH-A-3 | 8.45 | 45.76;45.78 | 98.08;97.87 | 90.76;90.43 | 82.17 |
|  | Middle | RH-M-1 | 7.36 | 45.63;45.64 | 98.19;97.80 | 91.24;90.25 | 83.18 |
|  |  | RH-M-2 | 6.88 | 45.62;45.63 | 98.11;97.75 | 90.88;90.04 | 83.19 |
|  |  | RH-M-3 | 8.47 | 45.66;45.66 | 98.05;97.71 | 90.62;89.84 | 83.03 |
|  | Basal | RH-B-1 | 8.79 | 45.81;45.81 | 98.06;97.77 | 90.72;90.09 | 82.30 |
|  |  | RH-B-2 | 8.55 | 45.86;45.86 | 98.32;98.05 | 91.63;91.03 | 82.42 |
|  |  | RH-B-3 | 7.46 | 45.85;45.87 | 98.20;97.84 | 91.28;90.40 | 81.82 |
| *R. fastigiata* | Apical | RF-A-1 | 9.32 | 45.83;45.85 | 98.23;97.87 | 91.37;90.51 | 72.89 |
|  |  | RF-A-2 | 8.34 | 45.93;45.93 | 98.19;97.78 | 91.17;90.14 | 73.40 |
|  |  | RF-A-3 | 8.67 | 45.73;45.76 | 98.16;97.89 | 91.02;90.46 | 72.84 |
|  | Middle | RF-M-1 | 7.78 | 45.65;45.66 | 98.18;97.77 | 91.13;90.11 | 73.36 |
|  |  | RF-M-2 | 7.26 | 45.66;45.66 | 98.09;97.81 | 90.80;90.19 | 73.09 |
|  |  | RF-M-3 | 7.19 | 45.76;45.76 | 98.24;97.66 | 91.41;89.78 | 73.3 |
|  | Basal | RF-B-1 | 8.02 | 45.90;45.91 | 98.15;97.81 | 91.00;90.25 | 73.46 |
|  |  | RF-B-2 | 7.69 | 45.91;45.92 | 97.93;97.79 | 90.13;90.04 | 72.08 |
|  |  | RF-B-3 | 6.50 | 45.90;45.91 | 98.09;97.77 | 90.86;90.12 | 72.97 |

Table S5 Gene-specific qRT-PCR primers for candidate UDP-glycosyltransferase genes

| Gene ID | Forward primer (5’ → 3’) | Reverse primer sequence (5’ → 3’) |
| --- | --- | --- |
| *RcUGT24044* | TCGTCATTGGAGGGGATTTCAG | TGAAAAACTGCTCGGCGAAC |
| *RcUGT21426* | CGACTTGCCCACGTTTATGAG | ACGTTGCGATTTCCTTGTGC |
| *RcUGT5914* | TGCGCAGAGACCACAAAAAC | TGGTGTTTCTGTGCTTTGGG |
| *RcUGT4379* | TTTCAATCATGGCCGTGCAG | AACCCGCAGTCAAGAAAACG |
| *RcUGT13191* | TGTCCATCAGCACGCAAAAC | ACGTGCAGCTTCCAAACATG |
| *RcUGT1732* | AAGCTCCAACTATCCGCACTAG | TCCGCGCTGTCAATTGATTC |
